# Grazing lawns and overgrazing in frequently grazed grass communities

**DOI:** 10.1002/ece3.9268

**Published:** 2022-09-13

**Authors:** Gareth P. Hempson, Catherine L. Parr, Caroline E. R. Lehmann, Sally Archibald

**Affiliations:** ^1^ Centre for African Ecology, School of Animal, Plant and Environmental Sciences University of the Witwatersrand Johannesburg South Africa; ^2^ South African Environmental Observation Network (SAEON), Ndlovu Node Phalaborwa South Africa; ^3^ Department of Earth, Ocean & Ecological Sciences University of Liverpool Liverpool UK; ^4^ Department of Zoology & Entomology University of Pretoria Pretoria South Africa; ^5^ School of GeoSciences University of Edinburgh Edinburgh UK; ^6^ Tropical Diversity Royal Botanic Garden Edinburgh Edinburgh UK

**Keywords:** degradation, environmental constraints, grass traits, growth forms, palatability, species composition

## Abstract

Frequent grazing can establish high forage value grazing lawns supporting high grazer densities, but can also produce overgrazed grass communities with unpalatable or low grass basal cover, supporting few grazers. Attempts to create grazing lawns via concentrated grazing, with a goal to increase grazer numbers, are thus risky without knowing how environmental conditions influence the likelihood of each outcome. We collected grass species and trait data from 33 frequently grazed grass communities across eastern South Africa (28 sites) and the Serengeti National Park, Tanzania (five sites), covering wide rainfall (336–987 mm year^−1^) and soil (e.g., 44%–93% sand) gradients. We identified four grass growth forms using hierarchical clustering on principal components analyses of trait data and assessed trait–environment and growth form–environment relationships using fourth corner and principal components analyses. We distinguished two palatable grass growth forms that both attract yet resist grazers and comprise grazing lawns: (1) “lateral attractors” that spread vegetatively via stolons and rhizomes, and (2) “tufted attractors” that form isolated tufts and may have alternate tall growth forms. By contrast, (3) tough, upright, tufted “resisters,” and (4) “avoiders” with sparse architectures or that grow appressed to the soil surface, are of little forage value and avoided by grazers. Grazing lawns occurred across a wide range of conditions, typically comprising lateral attractor grasses in drier, sandy environments, and tufted attractor grasses in wetter, low‐sand environments. Resisters occurred on clay‐rich soils in mesic areas, while avoiders were widespread but scarce. While grazing lawns can be established under most conditions, monitoring their composition and cover is important, as the potential for overgrazing seems as widely relevant. Tufted attractor‐dominated lawns appear somewhat more vulnerable to degradation than lateral attractor‐dominated lawns. Increased avoider and resister abundance both reduce forage value, although resisters may provide better soil protection.

## Graphical abstract

Frequent grazing can convert grass communities into grazing lawns or into overgrazed areas, with strongly opposing consequences for the number of grazers the region can support. By examining the diversity of grass life histories and species composition in frequently grazed areas across broad rainfall and soil gradients, we find that grazing lawns can establish under most conditions, but that their form and resilience vary. Importantly, the potential for overgrazing is also widespread.

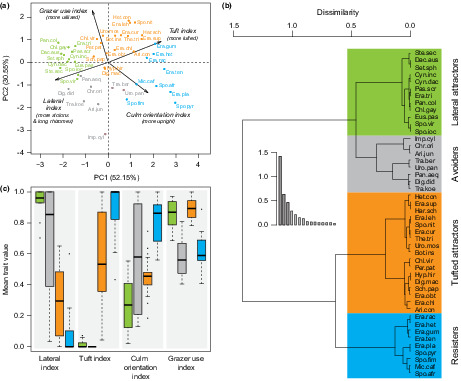

## 1 INTRODUCTION

Grazing lawns are short‐grass communities with dense leafy swards that provide high‐quality forage for grazers (McNaughton, 1984). While high forage digestibility and rapid intake rates attract grazers to lawns (Verweij et al., 2006), lawns also require regular grazing to prevent taller grass species from invading and outcompeting short‐statured lawn species for light (Hempson et al., 2019; McIvor et al., 2005; Waldram et al., 2008). Frequent grazing is thus essential to establish and maintain grazing lawns (McCauley et al., 2018; McNaughton, 1984). However, frequent grazing can also lead to the loss of grass basal cover and an increased abundance of annual species with sparse architectures with low forage value (Kelly & Walker, 1976; McNaughton, 1983; O'Connor, 1994). This “overgrazing” can result in increased bare ground, soil erosion, and run‐off and can be irreversible on human management timescales (van de Koppel et al., 1997). Thus while frequent grazing can create grazing lawns with high‐quality forage for grazers under some environmental conditions (Mislevy et al., 1982), it can also lead to overgrazing and degradation of the grazing resource (Illius & O'Connor, 1999). This poses a problem for conservation and rangeland practitioners who seek to increase grazer numbers by creating grazing lawns in the systems they manage, as a critical question remains unanswered: “when does frequent grazing produce grazing lawns, and when does it lead to overgrazing?”

Whether frequent grazing produces lawns or overgrazed conditions is important because they have generally opposite feedbacks on grazer population densities. Grazing lawns are principally a wet season forage resource due to their low standing biomass and thus need to co‐occur with adequate dry season forage reserves (Fynn, 2012; Kleynhans et al., 2011; Verweij et al., 2006). Under these conditions, the benefits of lawns accrue largely via improved grazer recruitment rates, with pregnant and lactating females more rapidly regaining body condition lost during the dry season, with concomitant benefits to their offspring (Cingolani et al., 1998; Hempson, Illius, et al., 2015). By contrast, and to some extent by definition (Mysterud, 2006), overgrazing degrades grazing systems by reducing the number of animals an area can support, due to year‐round constraints on grazer nutrition from reduced forage quantity and possibly also forage quality (Ash et al., 1995; Illius & O'Connor, 1999). Consequently, without a clear understanding of the potential for an area to support grazing lawns, there is much risk in attempting to establish grazing lawns via promoting locally concentrated increases in grazing pressure, e.g., by fencing, water point manipulation, mowing and nutrient additions (Cromsigt & Olff, 2008), or fire‐herbivory feedbacks (Archibald et al., 2005; Donaldson et al., 2018).

The opposing feedbacks to grazer populations from grazing lawns vs. overgrazed areas reflect differences in the amount and quality of grass forage. While forage quantity is determined in part by the extent of grass cover (i.e., versus bare ground), the traits and life histories of grasses are fundamental to shaping the quantity and quality of the grazing resource (Archibald et al., 2019; Coughenour, 1985). Viewed through a potential grazing event, grasses have trait syndromes that determine: (1) the likelihood of them being grazed (i.e., attractance‐avoidance), (2) how much and which plant parts can be consumed (i.e. resistance), and (3) how and how well they recover after being grazed (i.e., tolerance; Archibald et al., 2019). Grazing lawn grass species by definition are attractive to grazers and have their leaves consumed, so to persist in a community they also require trait combinations that allow them to resist grazers and minimize the loss of critical tissues and/or that allow them to tolerate grazing and recover rapidly through repeat grazing events. For example, classic grazing lawn grass species spread laterally along the soil surface via stolons, which protects their meristems from grazers, while simultaneously producing a leafy canopy with highly concentrated forage biomass that is accessible to grazers (McNaughton, 1979, 1984). By contrast, grasses that remain in overgrazed areas are expected to avoid being grazed, typically by having sparse architectures that provide little grazing value (Tefera et al., 2010). Alternately, grasses with tough leaves and stems are likely to be both strongly resistant to and hence avoided by grazers, such that their dominance reduces the grazing value of a grass community, yet without an increase in bare ground and the risk of erosion typically associated with overgrazing (Bouchenak‐Khelladi et al., 2020; O'Reagain, 1993).

While frequent grazing is required to create and maintain grazing lawns, it is likely that some environmental contexts will be more conducive to supporting grazing lawns than others. Grazing lawns appear to have coevolved with grazers (McNaughton, 1984), suggesting that lawns should be most prevalent at intermediate rainfall (c. 400–850 mm year^−1^) where grazer densities are typically highest in African ecosystems (Archibald & Hempson, 2016; Hempson, Archibald, et al., 2015). This is supported by the positive relationship between grass productivity and rainfall (Milchunas & Lauenroth, 1993; O'Connor et al., 2001), such that grass regrowth at low rainfall may be too low or infrequent to allow lawns to persist, while at high rainfall any lapse in grazing pressure increases the risk of tall grasses invading and shading out lawn species (Hempson et al., 2019; McNaughton, 1985; Verweij et al., 2006). Nonetheless, grazing lawns occur across a wide rainfall gradient from at least 400 mm year^−1^ (Mountain Zebra National Park, South Africa; Novellie & Gaylard, 2013) to over 1200 mm year^−1^ (Benue National Park, Cameroon; Verweij et al., 2006). Similarly, while soils do not appear to place absolute limits on grazing lawn distributions (Archibald et al., 2005; Stock et al., 2010), they are often associated with mineral or nutrient hotspots in a landscape (Gosling et al., 2012; Grant & Scholes, 2006). This may suggest that higher nutrient soils are better able to support replacement of grazed leaf tissues in lawns, but alternately, may simply reflect where grazers are more likely to concentrate and initiate lawns within a landscape (Hempson, Archibald, et al., 2015).

Frequent grazing that leads to a loss of grass cover and increased soil erosion is typically associated with drier regions. This higher vulnerability to “classic” overgrazing is likely due to the overall lower productivity of these regions that limits the potential for grasses to regrow and maintain lost or damaged tissues when regularly grazed and trampled. The generally higher rainfall stochasticity of these regions also favors annual grasses (Friedman, 2020), which with little competition for space or light are able to adopt sparse architectures with low grazing value (Archibald et al., 2019). Nonetheless, the association between drier regions and overgrazing may instead reflect a greater potential in these areas for grazer numbers to greatly exceed that which the available grazing can support (Illius & O'Connor, 2000). This can be a natural outcome of sporadic dry periods that strongly reduce primary productivity (Caughley & Gunn, 1993), which can be exacerbated by water provision and supplemental feeding that results in sedentary populations and increased grazer densities (Hempson et al., 2017; Sinclair & Fryxell, 1985; van de Koppel et al., 1997). Sandy, nutrient‐poor soils also appear particularly vulnerable to overgrazing (Owen‐Smith & Danckwerts, 1997; Tefera et al., 2010), possibly due to lower grass regrowth potential, although edaphic effects on grass productivity are likely contingent on rainfall (Dye & Spear, 1982). Lastly, frequent grazing might drive grass communities toward an undesirable state dominated by tough resister grasses, a pathway that is more likely to be associated with higher productivity and more stable growth conditions that favor the persistence of this long‐lived life history.

African ecosystems have a long evolutionary history of grazing and stand out globally for their high diversity of grazer species (Owen‐Smith, 2013) and should thus harbor a wide diversity of grazing‐adapted grasses (Cingolani et al., 2005; Milchunas & Lauenroth, 1993). Grazing lawns and overgrazing are both widely recognized features of African ecosystems, providing an ideal context to assess under what conditions frequent grazing is likely to produce grazing lawns and where the vulnerability to overgrazing is high. Here, we do this by assessing grass traits, life histories, and community composition in 33 frequently grazed sites distributed over a wide gradient of rainfall (336–987 mm year^−1^) and soils (e.g., 44%–93% sand) across South and East Africa. We predicted that: (1) grazing lawns with high cover of laterally spreading attractor species would be most prevalent at intermediate to high rainfall sites with higher nutrient soils, and (2) evidence of overgrazing such as bare ground and grasses with avoider life histories would be most prominent at drier and sandy, less productive sites.

## 2 METHODS

### 2.1 Study sites

**FIGURE 1:**
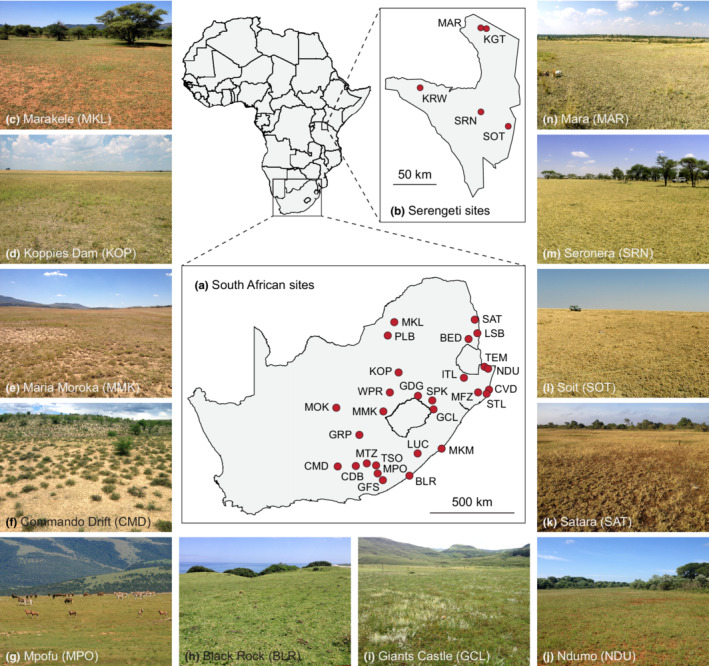
Locations of field sites in (a) South Africa and (b) Serengeti, with photos illustrating the wide diversity of environments in which regularly grazed sites occur (c–n). South African site name abbreviations: BED, Berg‐en‐Dal; BLR, Black Rock; CDB, Camdeboo; CMD, Commando Drift; CVD, Cape Vidal; GCL, Giants Castle; GDG, Golden Gate; GFS, Great Fish; GRP, Gariep Dam; ITL, Ithala; KOP, Koppies Dam; LSB, Lower Sabie; LUC, Luchaba; MFZ, iMfolozi; MKL, Marakele; MKM, Mkambati; MMK, Maria Moroka; MOK, Mokala; MPF, Mpofu; MTZ, Mountain Zebra; NDU, Ndumo; PLB, Pilanesberg; SAT, Satara; SPK, Spioenkop; STL, St Lucia; TEM, Tembe; TSO, Tsolwana; WPR, Willem Pretorius. Serengeti site name abbreviations: KGT, Kogatende; KRW, Kirawira; MAR, Mara; SOT, Soit; SRN, Seronera.

Grass communities were sampled at 28 sites in protected areas across the eastern half of South Africa between December 2014 and March 2015 (rainfall: 336–962 mm year^−1^), and five sites in the Serengeti National Park, Tanzania, in July 2016 (rainfall: 448–987 mm year^−1^; Figure 1, Table S1).

With the assistance of local managers, we identified sites that had been kept short (i.e., < 10 cm) continuously for a minimum of 10 years, predominantly by frequent grazing, although most sites had likely met these criteria for much longer. This ensured that: (1) light competition had not been a factor shaping community assembly, (2) grass communities had sufficient time to become representative of these conditions (Cromsigt & Olff, 2008; Donaldson et al., 2018), and that (3) disturbance regimes mostly constituted grazing and trampling by grazers. Note that 10 of the South African sites were located on airstrips or soccer fields within protected areas and may thus occasionally have been mowed to keep them short. However, in all cases these sites would predominantly have been kept short by grazing by indigenous grazer species. Furthermore, two South African sites were located in communal grazing areas in the buffer zone of protected areas and would predominantly have been grazed by cattle.

### 2.2 Sampling protocols

Sampling procedures characterized the grass species composition and grass growth forms at each site. Grass communities were sampled using 0.25 m^2^ quadrats distributed evenly through the frequently grazed habitat. Most sites had 30 quadrats but the minimum was 15 at one site where sampling was restricted by time. Full details of sampling areas and plot layout are provided in Table S1. Overall, the average distance between quadrats was ~12 m (range: 8–15 m).

All grass species occurring within a quadrat were identified in the field and verified at the National Herbarium in Pretoria, South Africa. For each grass species within a quadrat, we recorded percentage aerial cover, median leaf table height (mm), culm orientation (lateral, decumbent, geniculate, or upright), stolons (present or absent), and rhizomes (absent, short or long). Leaf table height was assessed visually as the approximate 80th quantile of leaf biomass, with the main bulk of the leaf canopy occurring below this height (Wigley et al., 2020). We classified short rhizomes as those that incrementally allowed an individual to expand the size of its base, forming a tuft, and long rhizomes as those facilitating the establishment of new ramets with spatially separate aboveground biomass. Percentage bare ground in each quadrat was recorded, and whether grazer dung was present or not. All data were collected by the same observer throughout the study.

### 2.3 Environmental data

Soil samples were collected at the four corners of each site and analyzed for texture (percent sand, silt and clay), cations (K, Ca, Mg and Na), exchangeable acidity, and pH. Cation exchange capacity (CEC) was calculated for each soil sample. South African soil samples were analyzed at the Agricultural Research Council Institute for Soil, Climate and Water, in Pretoria, South Africa, and Serengeti soils were analyzed at the Sokoine University of Agriculture, Morogoro, Tanzania. Daily rainfall data were extracted from the Climate Prediction Center (CPC) Africa Rainfall Climatology Version 2.0 (ARC2) dataset and used to calculate mean annual rainfall for each site for the 30‐year period prior to the sampling date. Access to these data was obtained via the Columbia University International Research Institute for Climate and Society website (iri.columbia.edu).

### 2.4 Trait indices

Grass species at our sites persist under frequent grazing, and we sought to characterize the key life history attributes that enable this (i.e., avoidance‐attractance, resistance, or tolerance). Four trait indices were derived from field measurements: (1) culm orientation index, (2) lateral index, (3) tuft index, and (4) grazer use index. Culm orientation index was calculated as the species mean value after converting each species × quadrat culm orientation record to a numerical value as follows: lateral = 1, geniculate‐lateral = 2, geniculate or decumbent = 3, geniculate‐upright = 4, and upright = 5. The lateral index was calculated as the proportion of quadrat‐level records where a species had stolons or long rhizomes. Similarly, the tuft index was calculated as the proportion of quadrat‐level records where a species had a tufted base. To indicate the relative site‐level grazing preference of a species, we derived a grazer use index based on the assumption that maximum grazer use is experienced by species with heights close to the median site‐level leaf table height, via: (1) taking the ratio of the leaf table height for each species × quadrat to the overall site‐level median leaf table height, (2) for values >1 (i.e., where a species × quadrat is taller than the overall site median), taking the reciprocal of this value, and (3) calculating the overall species mean value across all quadrats × sites. Low grazer use index values are thus obtained for: (1) species that are usually substantially taller than the median leaf table height at a site and thus inferred to be accessible to but less‐utilized by grazers, and (2) species that are considerably shorter than the median leaf table height at a site that are inferred to be largely inaccessible to and thus little‐utilized by grazers. Returning to our assumption that maximum grazer use is experienced at median site‐level leaf table height, it is possible that selective grazing of an uncommon species at a site may reduce its height relative to the median and that its use may be underestimated by the index. We anticipate that this will be rare, however, as sites were selected for their high grazing pressure, which should reduce the potential for highly selective grazing.

### 2.5 Growth form classifications

Data were analyzed in R 4.0.3 (R Core Team, 2020). Hierarchical cluster analysis was used to partition all grass species occurring in >10 quadrats across all sites into life history strategies based on the four trait indices, using hierarchical clustering on principal components (“HCPC” function in the “FactoMineR” R package, hereafter *FactoMineR::HCPC*; Le et al., 2008). Accordingly, principal components analysis (PCA) of the species traits (with standardized range from 0 to 1) was performed prior to clustering using *FactoMineR::PCA*, minimizing the impact of covariance among traits on the clustering algorithm. Clustering proceeds in an agglomerative fashion using a Euclidian distance dissimilarity matrix of the PCA dimensions and Ward's method, grouping the most similar clusters until all species have been classified. Trait descriptions for each growth form were obtained using a v‐test, which compares within cluster trait values to the overall trait value. Trait values and personal knowledge of species characteristics were used to manually assign species occurring in 10 or fewer quadrats to growth form clusters (G. P. Hempson), as including these species in the formal clustering procedure tended to destabilize the clusters. All species life history strategy classifications and trait values are provided in Table S2.

### 2.6 Trait–environment relationships

Trait relationships with rainfall and soil conditions were assessed via fourth corner analyses using *mvabund::traitglm* (Wang et al., 2018). Analyses were restricted to the minimal set of species that together comprised 90% cover at a site (range: 2–12 species) and were thereafter scored as present/absent for fitting a model with binomial errors. The approach discards abundance information but captures the dominant species at a site while reducing challenges around identifying an appropriate error distribution. All four trait indices were included in the species × trait matrix throughout the analysis. Mean annual rainfall, percent sand, cation exchange capacity (CEC), and pH were fitted as environmental variables in the full model. A full subset of models with all environmental variables was fitted, and the most supported model identified using Akaike's Information Criterion corrected for small sample sizes (AICc; *MuMIn:AICc*; Bartoń, 2020). The significance of the overall trait–environment interactions was assessed using an ANOVA with 999 resampling iterations performed via PIT‐trap (probability integral transform residuals) block resampling (*mvabund::anova*).

### 2.7 Life history strategy–environment relationships

Life history strategy–environment relationships were assessed using contour plots: PCA (*FactoMineR::PCA*) was used to extract the first two axes capturing environmental variation among sites (mean annual rainfall, percent sand, CEC, and pH), with the percentage cover of each grass growth form plotted on the *z*‐axis. All grass species occurring at a site were included in the analysis.

### 2.8 Percentage bare ground

Percentage bare ground is a key variable used to assess whether a system is overgrazed or degraded and should increase with grazing pressure in systems prone to overgrazing, while the opposite should be true in systems with the potential to develop grazing lawns. Therefore, to test whether the dominant grass growth form can predict degradation risk we ran two multiple linear regressions, on communities dominated by (1) lateral‐spreading grasses and (2) tufted grasses (see growth form classification results below: “lateral attractors” and “tufted attractors”). The global regression model for each analysis included the interaction effects of mean annual rainfall, percentage sand, and proportion of quadrats with dung present (as a proxy for site‐level grazing pressure) on percentage bare ground at each site, with AICc used to identify the best model among all nested models.

## 3 RESULTS

### 3.1 Growth form classifications

**FIGURE 2:**
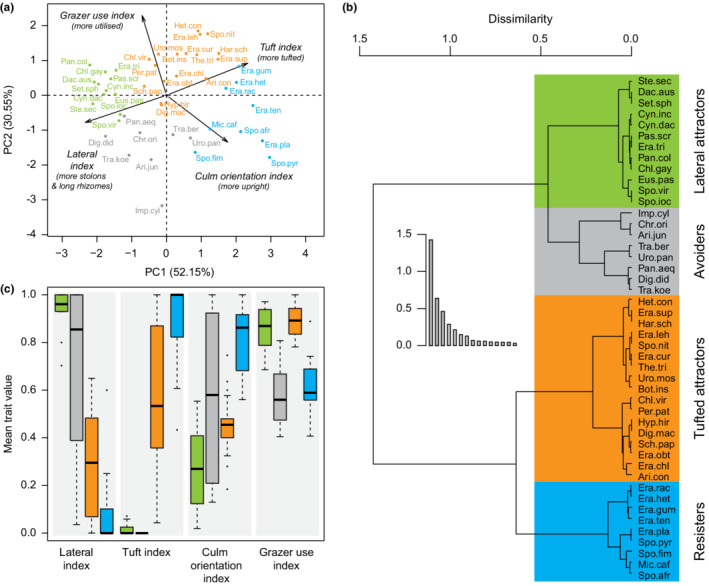
Life history strategy classification of grasses occurring in frequently grazed sites in South Africa and the Serengeti, Tanzania. Life history strategies were classified based on four traits (lateral, tuft, culm orientation, and grazer use indices) that were first subjected to principal components analysis (a), with agglomerative hierarchical clustering then performed on these principal components (b). The resulting tree was cut to produce four ecologically meaningful groups (lateral attractors, avoiders, tufted attractors, and resisters), which differ in the mean and variability of their trait values (c). Species abbreviations are the derived from the first three letters of genus and species, with full names provided in Table S2.

A total of 88 grass species were recorded during the study (Table S2). Four ecologically interpretable grass life history strategies were identified among the 46 species included in the HCPC analysis (Figure 2). The lateral and tuft indices had the highest loadings on the first axis of the trait PCA (PC1 explained 52.15% of variance; Figure 2a, Table S3), and the highest split in the clustering tree thus broadly grouped species by whether they frequently had stolons and/or long rhizomes, or whether they typically had a tufted base (Figure 2a,b). The grazer use index had the highest loading on PC2 (30.55% of variance) and was influential in separating species with higher lateral index scores into two groups, which we refer to as “lateral attractors” and “avoiders.” For the lateral attractors, the v‐tests revealed that group means for all four trait indices differed significantly from the overall means, with higher lateral (*t*_(45)_ = 4.491, *p* < .001) and grazer use (*t*_(45)_ = 1.988, *p* = .047) index values, and lower culm orientation (*t*_(45)_ = −3.300, *p* < .001) and tuft (*t*_(45)_ = −3.723, *p* < .001) index values (Figure 2c; Table S4). By contrast, the avoiders had significantly low grazer use (*t*_(45)_ = −3.625, *p* < .001) and tuft (*t*_(45)_ = −2.994, *p* = .003) index values.

The remaining species with higher tuft index values were separated into two groups, “tufted attractors” and “resisters,” based on differences in their grazer use and culm orientation index values. Tufted attractors had high grazer use (*t*_(45)_ = 3.487, *p* < .001) and tuft (*t*_(45)_ = 2.562, *p* = .01) index values, and low lateral index values (*t*_(45)_ = −2.664, *p* = .007) compared with the overall means. Resisters had high culm orientation (*t*_(45)_ = 3.914, *p* < .001) and tuft (*t*_(45)_ = 3.863, *p* < .001) index values, and low lateral (*t*_(45)_ = −3.211, *p* = .001) and grazer use (*t*_(45)_ = −2.980, *p* = .003) index values. Note that although tufted attractor grasses generally had high tuft and low lateral index values, there is much variability within traits in this group, but all are apparently highly preferred.

### 3.2 Trait–environment relationships

**FIGURE 3:**
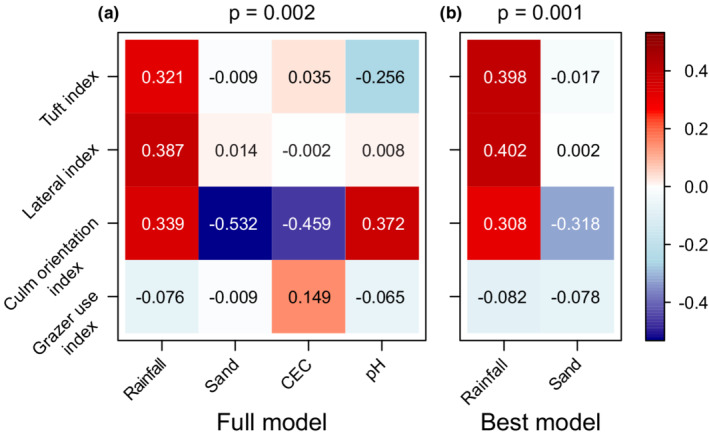
Fourth corner analyses to assess relationships between grass traits and rainfall and soil attributes. The full model included all 33 study sites and four environmental variables (mean annual rainfall, percent sand, cation exchange capacity, and pH) in the site × environment matrix. All four grass traits (lateral, tuft, culm orientation, and grazer use indices) were included in the species × trait matrix for all models. The species × site matrix contained the minimal set of species that together comprised 90% cover at a site and which were then scored as present or absent for fitting a binomial error distribution. *p*‐values represent support for an overall trait‐environment effect in the model. Coloring represents the coefficient values for specific trait–environment associations estimated in the model.

Overall, grass traits showed the strongest associations with rainfall and percent sand, which were retained in the best model (*p* = .001; Figure 3b), although the trait–environment coefficients in the full model (*p* = .002; ΔAIC = 8.256 from best model; Figure 3a) also indicates clear links between grass traits and soil CEC and pH, respectively. The positive association between rainfall and the tuft, lateral and culm orientation indices in both the full and best models indicates that the incidence of tufted species, laterally spreading species, and species with more upright culms increased in wetter areas.

By contrast, there was strong evidence for a higher incidence of grasses with more prostrate culm orientations in areas with sandy soils. Although soils with high CEC also tend to have higher pH, the full model indicates that culm orientation is more upright on the higher pH soils and more prostrate on soils with higher CEC (Figure 3a). There is also evidence that the incidence of tufted species is higher on more acidic soils. The grazer use index showed little association with rainfall, sand, or pH and had a weak positive association with soil CEC.

### 3.3 Life history strategy–environment relationships

**FIGURE 4:**
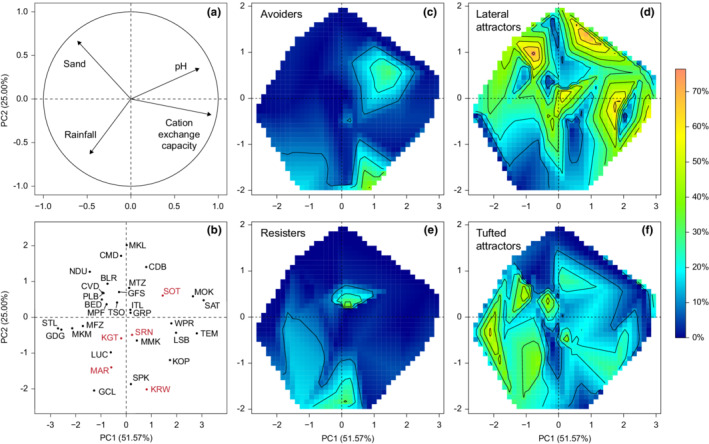
Life history strategy cover along environmental gradients. Sites were ordinated using principal components analysis of four environmental variables (mean annual rainfall, percent sand, cation exchange capacity, and pH), with the variable loadings on the first two principal components shown in (a), and site positions on these axes shown in (b). the site‐level percentage cover of the avoider (c), lateral attractor (d), resister (e), and tufted attractor (f) life history strategies was interpolated across site locations on PC1 and PC2. Contour intervals represent a 10% increase in cover, with dark blue representing 0% cover. Site name abbreviations correspond to Figure 1, with Serengeti sites shown in red in (b).

The 33 study sites spanned a diverse range of environmental conditions and were fairly evenly distributed across the first two principal component axes (Figure 4a,b). The first axis accounted for 51.57% of variation among sites (Table S5) and represents a classic “rich savanna‐poor savanna” gradient from areas with basic, high CEC soils occurring in drier, less sandy regions, to areas that have sandier, less fertile soils and that tend to be wetter. The second axis accounted for 25.00% of variation among sites, and distinguishes sites with sandier, drier, higher pH soils from wetter sites with more clay‐rich soils and higher CEC. Interpolation of the site‐level proportional cover of each life history strategy across PC1 and PC2 shows that the lateral attractor (Figure 4d) and tufted attractor (Figure 4f) life history strategies are more abundant than avoiders (Figure 4c) and resisters (Figure 4e), and occur across a wider range of environmental conditions. Lateral attractors had >30% cover at a site under a wide range of environmental conditions, but were particularly abundant in sandy and drier regions and do not appear to be strongly influenced by soil fertility (CEC and pH). The one region of environmental space where lateral attractors had low abundance, however, was in high rainfall areas with acidic soils, which instead was dominated by tufted attractor species. Resisters were most abundant in high rainfall areas on soils with low sand content and higher CEC values, but also showed a peak at the Ithala site, which had intermediate rainfall and percent sand values. Avoiders were generally scarce, exceeding 30% cover only at the Kirawira site in the Serengeti, where *Chrysochloa orientalis* was abundant. Overall, the broad patterns evident in life history strategy distributions across environmental axes are also clearly subject to many exceptions.

### 3.4 Percentage bare ground

**FIGURE 5:**
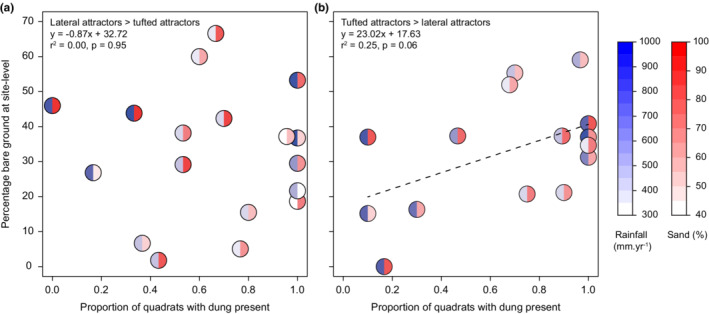
Bare ground in relation to dung abundance at sites where (a) lateral attractors are more abundant than tufted attractor growth forms, and (b) vice versa. Linear regression showed no relationship between bare ground and dung abundance on lateral attractor‐dominated sites, but a marginally significant positive relationship on tufted attractor‐dominated sites. Rainfall and sand have no effect on these relationships, as illustrated by blue and red shading, respectively.

The average percentage bare ground across all sites was 32.5% (±3.0 SE) and did not differ between sites dominated by lateral (32.2%) and tufted (33.0%) attractor grasses (*F*_1,31_ = 0.019, *p* = .891). In lateral attractor‐dominated sites, there was no evidence for grazing pressure, rainfall, or sand influencing the percentage bare ground, with the intercept only model being preferred (Table S6). There was some evidence that grazer utilization may increase the percentage bare ground in tufted attractor‐dominated sites: the best model included only a positive and marginally significant effect of dung abundance on percentage bare ground (*F*_1,13_ = 4.312, *p* = .06; Figure 5; Table S7); however, the simpler intercept only model received similar support (ΔAICc = 1.115).

## 4 DISCUSSION

Frequently grazed areas display considerable diversity in grass traits, growth forms, and community composition (Figure 2). Counter to our predictions, (1) we observed grazer attractor grasses that comprise grazing lawns across all rainfall and soil conditions, with tufted attractors being more prevalent at high rainfall than lateral attractors, and (2) the prevalence of avoider grasses and bare ground—our indicators of overgrazing—was not clearly linked to environmental conditions (Figure 4). The potential for grazing lawn establishment but also for overgrazing thus seems widespread. However, the diversity and distribution of grass growth forms we observe across frequently grazed sites should be useful in assessing the risk/benefit trade‐off of using concentrated grazing to establish grazing lawns in different environments, as discussed below.

### 4.1 Grass life history strategies and their environmental relationships

The four grass growth forms we identified from trait clustering can be distinguished based on how they enable grasses to attract vs. avoid, resist or tolerate frequent grazing (Archibald et al., 2019). The lateral attractor cluster aligns most closely with “classic” grazing lawn species, showing high rates of lateral spread via stolons and rhizomes, and often having prostrate flowering culms (McNaughton, 1984). This protects their stems or stolons, buds, and roots below the bite depth of grazers (Coughenour, 1985), leaving only highly digestible leaf material accessible to grazers. Lateral attractors were dominant at heavily grazed sites across a wide range of environmental conditions, although they tended to be replaced by tufted attractors in higher rainfall areas with more fine‐textured soils.

The tufted attractor cluster includes a range of grass life histories. Many of these species would be considered “generalist tolerators” (Archibald et al., 2019), as they are able to adopt taller, upright growth forms in fire‐prone communities with lower grazing pressure (e.g., *Themeda triandra*, *Heteropogon contortus*, and *Hyparrhenia hirta*). Resprouting from underground stored reserves is thought to be central to the ability of these species to tolerate repeated fires and/or grazing (Coughenour, 1985; Ripley et al., 2015). As such, the association we found between tufted attractors and higher nutrient, higher rainfall soils are expected as these soils would best enable compensatory regrowth required for this strategy to be successful. Among the other species in the tufted attractor cluster are short‐statured species such as *Sporobolus nitens*, which does not have an alternate tall growth form. This species is commonly associated with soils with high sodium concentrations (Bailey & Scholes, 1997), and frequent grazing in these sites (Grant & Scholes, 2006)—driven by herbivore sodium demands—is likely important to maintain access to light for these short‐statured grasses. Sodium‐enriched soils are probably predisposed to achieving the frequent grazing necessary to establish and maintain grazing lawns, while the role of silica, which was not assessed in this study, is also an intriguing avenue for future research given its varied roles in plant defense (e.g., Hummel et al., 2011), as a grass growth promotor (McNaughton et al., 1985), and the variation in its availability in response to parent material, soil texture, pH, and rainfall (Quigley et al., 2017).

The resister and avoider grass growth form clusters both had low grazer use values, but likely for divergent reasons. Resister grasses have tufted, upright, and often stemmy architectures and have tough leaves with high C:N ratios and are very strongly rooted (e.g. *Eragrostis plana* and *Sporobolus pyramidalis*). Grazers are thus typically unable to remove much material from these grasses, likely resulting in them being avoided and growing taller than other grasses in frequently grazed areas. Thus, while the grazing value of the grass community is reduced when these grasses increase in abundance, a consolatory factor is that the soil surface remains fairly well protected. The avoider cluster included a variety of grass morphologies, for example, species that avoid grazing by growing tightly appressed to the soil surface (e.g., *Chrysopogon orientalis*, *Tragus beteronianus*), and species with sparse architectures that are of little forage value to grazers (e.g., *Panicum aequinerve*). While our analyses identify an intuitive set of growth form clusters, the counterintuitive classification of some species (e.g., *Imperata cyclindrica* as an avoider vs. resister, *Microchloa caffra* as a resister vs. avoider) suggests that including a wider range of traits (e.g., bulk density, leaf C:N) may result in clearer patterns. More generally, the many exceptions in the distribution of grass life history strategies across environmental gradients suggest that specific site‐level contingencies and histories likely also shape grass community composition, including variation in grazing pressure above that required to meet the study site inclusion criteria.

### 4.2 Grazing value and degradation

Our data show that grazing lawns can comprise both mat‐forming stoloniferous grasses and also tufted species that may not spread laterally (Figure 2; Cromsigt & Olff, 2008), and that these grasses can occur at over 50% cover under a broad range of rainfall and soil conditions (Figure 4). While the high leaf bulk densities of these growth forms are important for attracting grazers, the percentage cover of these growth forms is also fundamental to grazing value, because sward continuity has a large effect on intake rates given that bite depths will be relatively shallow (Murray & Illius, 2000). Regardless of which “grazing attractor” growth form dominates a lawn, frequent grazing is important to prevent shifts in growth form (e.g., by generalist tolerators) or invasion by new species (e.g. resisters) that result in a taller, more stemmy, and less palatable sward as occurs when grazing pressure lapses (Hempson et al., 2019; McCauley et al., 2018; Verweij et al., 2006). The positive feedback between grazers and sward quality, which is fundamental to grazing lawn dynamics (Coughenour, 1985; Hempson, Archibald, et al., 2015; McNaughton, 1984), is thus not restricted to stoloniferous, mat‐forming grasses, and lawns can thus be considered more generally as “short‐statured, grazer‐dependent grass communities that persist via positive feedbacks with grazers”.

Recognizing that lawns can be dominated by tufted attractors is important, because many species are considered classic “Decreaser” species (e.g., *Themeda triandra*) that are typically eliminated by heavy grazing and replaced by undesirable “Increaser II” or “Invader” species (Dyksterhuis, 1949; Trollope et al., 1989). This has motivated widespread rotational grazing management practices, which seek to provide plants a rest period from grazing during the growing season (Briske et al., 2008), opposite to the conditions necessary for grazing lawn establishment and maintenance. This low risk strategy eschews the potential benefits of incorporating grazing lawns as a wet season resource in a grazing system that also includes adequate dry season forage reserves (Fynn, 2012; Illius & O'Connor, 1999; Yoganand & Owen‐Smith, 2014). An important consideration, however, is that the close link between rainfall and primary production on lawns necessarily means that the duration and timing of their forage value mirrors the pattern of rainfall events (Bonnet et al., 2010), which may diminish their overall utility in more stochastic, drier regions.

While our survey of frequently grazed grass communities suggests that grazing lawns can be established under a wide range of environmental conditions, the risk of degradation cannot be ignored. Grazing lawn degradation can take at least three forms: (1) undergrazing, allowing the encroachment of taller, less palatable species or growth forms, (2) transitions to unpalatable resister and/or avoider species, and (3) increases in bare ground. Our results suggest that grazing lawns dominated by tufted attractor species may be most susceptible to each of these forms of degradation. Firstly, many tufted attractors have alternate, tall growth forms, and may be quicker to transition to more stemmy swards during any lapse in grazing frequency. Second, the environmental overlap with resister species in mesic areas with clay‐rich soils suggests a greater vulnerability to being invaded by these species (Figure 4), although further research is required to understand whether resisters invade lawns when under‐ vs. overgrazed. Lastly, the percentage bare ground in grazing lawns dominated by tufted attractors appears more closely linked to grazing pressure than in lawns dominated by lateral attractors (Figure 5). Nonetheless, it would seem logical to expect that for all grazing lawns a level of grazing and associated trampling pressure exists that would result in severe degradation, and hence that both minimum and maximum grazer use thresholds exist for all grazing lawn communities.

## 5 CONCLUSIONS AND MANAGEMENT CONSIDERATIONS

While it appears possible to form grazing lawns across a broad range of environmental conditions, further research is needed to bound the window of optimal grazing pressure for grazing lawn establishment and maintenance—both across environmental gradients, and for lawns comprising lateral vs. tufted attractor growth forms. The grazing value of lawns derives from the rapid replacement of consumed leaf material (McNaughton, 1985), a process that is closely linked to rainfall patterns (Bonnet et al., 2010), which may thus enhance their utility in wetter regions. Key considerations when seeking to incorporate lawns into a grazing system include recognizing that lawns comprise a wet season resource that requires frequent grazing during the growth season, which may be at odds with management practices such as some forms of rotational grazing. Monitoring the growth form and species composition of grazing lawns and the extent of bare ground during the wet season are key parameters for understanding the grazing value trajectory of a lawn, and hence whether grazing pressures should be adjusted. With due consideration of the degradation risks, conservation and rangeland practitioners who make careful use of concentrated grazing to establish grazing lawns stand to benefit from an undervalued grazing resource representing millions of years of coevolution between grazers and grasses.

## AUTHOR CONTRIBUTIONS

**Catherine L. Parr:** Conceptualization (equal); funding acquisition (equal); methodology (equal); writing – review and editing (equal). **Sally Archibald:** Conceptualization (equal); formal analysis (equal); funding acquisition (lead); investigation (equal); methodology (equal); project administration (lead); resources (lead); writing – review and editing (equal). **Caroline E. R. Lehmann:** Formal analysis (supporting); investigation (equal); writing – review and editing (equal). **Gareth P. Hempson:** Conceptualization (equal); data curation (lead); formal analysis (lead); investigation (lead); methodology (equal); visualization (lead); writing – original draft (lead).

## CONFLICT OF INTEREST

The authors declare that they have no competing interests.

## Supporting information


Tables S1–S7

## Data Availability

Data are available from Dryad: https://doi.org/10.5061/dryad.wstqjq2q6.

## References

[ece39268-bib-0001] Archibald, S. , Bond, W. J. , Stock, W. D. , & Fairbanks, D. H. K. (2005). Shaping the landscape: Fire–grazer interactions in an African savanna. Ecological Applications, 15(1), 96–109.

[ece39268-bib-0002] Archibald, S. , & Hempson, G. P. (2016). Competing consumers: Contrasting the patterns and impacts of fire and mammalian herbivory in Africa. Philosophical Transactions of the Royal Society, B: Biological Sciences, 371(1703), 20150309. 10.1098/rstb.2015.0309 PMC4978867 27502374

[ece39268-bib-0003] Archibald, S. , Hempson, G. P. , & Lehmann, C. (2019). A unified framework for plant life‐history strategies shaped by fire and herbivory. New Phytologist, 224(4), 1490–1503. 31177547 10.1111/nph.15986

[ece39268-bib-0004] Ash, A. J. , McIvor, J. G. , Corfield, J. P. , & Winter, W. H. (1995). How land condition alters plant‐animal relationships in Australia's tropical rangelands. Agriculture, Ecosystems & Environment, 56(2), 77–92.

[ece39268-bib-0005] Bailey, C. L. , & Scholes, M. C. (1997). Comparative patterns of sodium accumulation in leaves of selected savanna species growing on sodic and nonsodic soils. South African Journal of Plant and Soil, 14(3), 103–106.

[ece39268-bib-0006] Bartoń, K. (2020). MuMIn: Multi‐model inference . R package version 1.43.17. https://CRAN.R‐project.org/package=MuMIn

[ece39268-bib-0007] Bonnet, O. , Fritz, H. , Gignoux, J. , & Meuret, M. (2010). Challenges of foraging on a high‐quality but unpredictable food source: The dynamics of grass production and consumption in savanna grazing lawns. Journal of Ecology, 98(4), 908–916.

[ece39268-bib-0008] Bouchenak‐Khelladi, Y. , February, E. C. , Verboom, G. A. , & Boucher, F. C. (2020). C_4_ grass functional traits are correlated with biotic and abiotic gradients in an African savanna. Plant Ecology, 221(4), 241–254.

[ece39268-bib-0009] Briske, D. D. , Derner, J. D. , Brown, J. R. , Fuhlendorf, S. D. , Teague, W. R. , Havstad, K. M. , Gillen, R. L. , Ash, A. J. , & Willms, W. D. (2008). Rotational grazing on rangelands: Reconciliation of perception and experimental evidence. Rangeland Ecology & Management, 61(1), 3–17.

[ece39268-bib-0010] Caughley, G. , & Gunn, A. (1993). Dynamics of large herbivores in deserts: Kangaroos and caribou. Oikos, 67, 47–55.

[ece39268-bib-0011] Cingolani, A. M. , Anchorena, J. , & Collantes, M. B. (1998). Landscape heterogeneity and long‐term animal production in Tierra del Fuego. Rangeland Ecology & Management, 51(1), 79–87.

[ece39268-bib-0012] Cingolani, A. M. , Noy‐Meir, I. , & Díaz, S. (2005). Grazing effects on rangeland diversity: A synthesis of contemporary models. Ecological Applications, 15(2), 757–773.

[ece39268-bib-0013] Coughenour, M. B. (1985). Graminoid responses to grazing by large herbivores: Adaptations, exaptations, and interacting processes. Annals of the Missouri Botanical Garden, 72(4), 852–863.

[ece39268-bib-0014] Cromsigt, J. P. , & Olff, H. (2008). Dynamics of grazing lawn formation: An experimental test of the role of scale‐dependent processes. Oikos, 117(10), 1444–1452.

[ece39268-bib-0015] Donaldson, J. E. , Archibald, S. , Govender, N. , Pollard, D. , Luhdo, Z. , & Parr, C. L. (2018). Ecological engineering through fire‐herbivory feedbacks drives the formation of savanna grazing lawns. Journal of Applied Ecology, 55(1), 225–235.

[ece39268-bib-0016] Dye, P. J. , & Spear, P. T. (1982). Effects of bush clearing and rainfall variability on grass yield and composition in south‐West Zimbabwe. Zimbabwe Journal of Agricultural Research, 20, 103–118.

[ece39268-bib-0017] Dyksterhuis, E. J. (1949). Condition and management of range land based on quantitative ecology. Journal of Range Management, 2(3), 104–115.

[ece39268-bib-0018] Friedman, J. (2020). The evolution of annual and perennial plant life histories: Ecological correlates and genetic mechanisms. Annual Review of Ecology, Evolution, and Systematics, 51, 461–481.

[ece39268-bib-0019] Fynn, R. W. (2012). Functional resource heterogeneity increases livestock and rangeland productivity. Rangeland Ecology & Management, 65(4), 319–329.

[ece39268-bib-0020] Gosling, C. M. , Cromsigt, J. P. , Mpanza, N. , & Olff, H. (2012). Effects of erosion from mounds of different termite genera on distinct functional grassland types in an African savannah. Ecosystems, 15(1), 128–139. 25983634 10.1007/s10021-011-9497-8 PMC4423950

[ece39268-bib-0021] Grant, C. C. , & Scholes, M. C. (2006). The importance of nutrient hot‐spots in the conservation and management of large wild mammalian herbivores in semi‐arid savannas. Biological Conservation, 130(3), 426–437.

[ece39268-bib-0022] Hempson, G. P. , Archibald, S. , & Bond, W. J. (2017). The consequences of replacing wildlife with livestock in Africa. Scientific Reports, 7(1), 1–10. 29222494 10.1038/s41598-017-17348-4 PMC5722938

[ece39268-bib-0023] Hempson, G. P. , Archibald, S. , Bond, W. J. , Ellis, R. P. , Grant, C. C. , Kruger, F. J. , Kruger, L. M. , Moxley, C. , Owen‐Smith, N. , Peel, M. J. , & Smit, I. P. (2015). Ecology of grazing lawns in Africa. Biological Reviews, 90(3), 979–994. 25231416 10.1111/brv.12145

[ece39268-bib-0024] Hempson, G. P. , Archibald, S. , Donaldson, J. E. , & Lehmann, C. E. (2019). Alternate grassy ecosystem states are determined by palatability–flammability trade‐offs. Trends in Ecology & Evolution, 34(4), 286–290. 30791976 10.1016/j.tree.2019.01.007

[ece39268-bib-0025] Hempson, G. P. , Illius, A. W. , Hendricks, H. H. , Bond, W. J. , & Vetter, S. (2015). Herbivore population regulation and resource heterogeneity in a stochastic environment. Ecology, 96(8), 2170–2180. 26405742 10.1890/14-1501.1

[ece39268-bib-0026] Hummel, J. , Findeisen, E. , Südekum, K. H. , Ruf, I. , Kaiser, T. M. , Bucher, M. , Clauss, M. , & Codron, D. (2011). Another one bites the dust: Faecal silica levels in large herbivores correlate with high‐crowned teeth. Proceedings of the Royal Society B: Biological Sciences, 278(1712), 1742–1747. 10.1098/rspb.2010.1939 PMC3081769 21068036

[ece39268-bib-0027] Illius, A. W. , & O'Connor, T. G. (1999). On the relevance of nonequilibrium concepts to arid and semiarid grazing systems. Ecological Applications, 9(3), 798–813.

[ece39268-bib-0028] Illius, A. W. , & O'Connor, T. G. (2000). Resource heterogeneity and ungulate population dynamics. Oikos, 89(2), 283–294.

[ece39268-bib-0029] Kelly, R. D. , & Walker, B. H. (1976). The effects of different forms of land use on the ecology of a semi‐arid region in South‐Eastern Rhodesia. The Journal of Ecology, 64, 553–576.

[ece39268-bib-0030] Kleynhans, E. J. , Jolles, A. E. , Bos, M. R. , & Olff, H. (2011). Resource partitioning along multiple niche dimensions in differently sized African savanna grazers. Oikos, 120(4), 591–600.

[ece39268-bib-0031] Le, S. , Josse, J. , & Husson, F. (2008). FactoMineR: An R package for multivariate analysis. Journal of Statistical Software, 25(1), 1–18.

[ece39268-bib-0032] McCauley, D. J. , Graham, S. I. , Dawson, T. E. , Power, M. E. , Ogada, M. , Nyingi, W. D. , Githaiga, J. M. , Nyunja, J. , Hughey, L. F. , & Brashares, J. S. (2018). Diverse effects of the common hippopotamus on plant communities and soil chemistry. Oecologia, 188(3), 821–835. 30099603 10.1007/s00442-018-4243-y

[ece39268-bib-0033] McIvor, J. G. , McIntyre, S. , Saeli, I. , & Hodgkinson, J. J. (2005). Patch dynamics in grazed subtropical native pastures in south‐East Queensland. Austral Ecology, 30(4), 445–464.

[ece39268-bib-0034] McNaughton, S. J. (1979). Grassland‐herbivore dynamics. In A. R. E. Sinclair & M. Norton‐Griffiths (Eds.), Serengeti: Dynamics of an ecosystem (pp. 46–81). University of Chicago Press.

[ece39268-bib-0035] McNaughton, S. J. (1983). Serengeti grassland ecology: The role of composite environmental factors and contingency in community organization. Ecological Monographs, 53(3), 291–320.

[ece39268-bib-0036] McNaughton, S. J. (1984). Grazing lawns: Animals in herds, plant form, and coevolution. The American Naturalist, 124(6), 863–886.

[ece39268-bib-0037] McNaughton, S. J. (1985). Ecology of a grazing system: The Serengeti. Ecological Monographs, 53, 291–320.

[ece39268-bib-0038] McNaughton, S. J. , Tarrants, J. L. , McNaughton, M. M. , & Davis, R. D. (1985). Silica as a defense against herbivory and a growth promotor in African grasses. Ecology, 66(2), 528–535.

[ece39268-bib-0039] Milchunas, D. G. , & Lauenroth, W. K. (1993). Quantitative effects of grazing on vegetation and soils over a global range of environments. Ecological Monographs, 63(4), 327–366.

[ece39268-bib-0040] Mislevy, P. , Mott, G. O. , & Martin, F. G. (1982). Effect of grazing frequency on forage quality and stolon characteristics of tropical perennial grasses. Proceedings of the Soil and Crop Science Society of Florida, 41, 77–83.

[ece39268-bib-0041] Murray, M. G. , & Illius, A. W. (2000). Vegetation modification and resource competition in grazing ungulates. Oikos, 89(3), 501–508.

[ece39268-bib-0042] Mysterud, A. (2006). The concept of overgrazing and its role in management of large herbivores. Wildlife Biology, 12(2), 129–141.

[ece39268-bib-0043] Novellie, P. , & Gaylard, A. (2013). Long‐term stability of grazing lawns in a small protected area, the mountain zebra National Park. Koedoe, 55(1), 1–7.

[ece39268-bib-0044] O'Connor, T. G. (1994). Composition and population responses of an African savanna grassland to rainfall and grazing. Journal of Applied Ecology, 31, 155–171.

[ece39268-bib-0045] O'Connor, T. G. , Haines, L. M. , & Snyman, H. A. (2001). Influence of precipitation and species composition on phytomass of a semi‐arid African grassland. Journal of Ecology, 89(5), 850–860.

[ece39268-bib-0046] O'Reagain, P. J. (1993). Plant structure and the acceptability of different grasses to sheep. Journal of Range Management, 46(3), 232–236.

[ece39268-bib-0047] Owen‐Smith, N. (2013). Contrasts in the large herbivore faunas of the southern continents in the late Pleistocene and the ecological implications for human origins. Journal of Biogeography, 40(7), 1215–1224.

[ece39268-bib-0048] Owen‐Smith, N. , & Danckwerts, J. E. (1997). Chapter 17: Herbivory. In R. M. Cowling , D. M. Richardson , & S. M. Pierce (Eds.), Vegetation of southern Africa (pp. 397–420). Cambridge University Press.

[ece39268-bib-0049] Quigley, K. M. , Donati, G. L. , & Anderson, T. M. (2017). Variation in the soil ‘silicon landscape’ explains plant silica accumulation across environmental gradients in Serengeti. Plant and Soil, 410(1), 217–229.

[ece39268-bib-0050] R Core Team . (2020). R: A language and environment for statistical computing. R Foundation for Statistical Computing. https://www.R‐project.org/

[ece39268-bib-0051] Ripley, B. , Visser, V. , Christin, P. A. , Archibald, S. , Martin, T. , & Osborne, C. (2015). Fire ecology of C3 and C4 grasses depends on evolutionary history and frequency of burning but not photosynthetic type. Ecology, 96(10), 2679–2691. 26649389 10.1890/14-1495.1

[ece39268-bib-0052] Sinclair, A. R. E. , & Fryxell, J. M. (1985). The Sahel of Africa: Ecology of a disaster. Canadian Journal of Zoology, 63(5), 987–994.

[ece39268-bib-0053] Stock, W. D. , Bond, W. J. , & van der Vijver, C. A. D. M. (2010). Herbivore and nutrient control of lawn and bunch grass distributions in a southern African savanna. Plant Ecology, 204(1), 15–27.

[ece39268-bib-0054] Tefera, S. , Dlamini, B. J. , & Dlamini, A. M. (2010). Changes in soil characteristics and grass layer condition in relation to land management systems in the semi‐arid savannas of Swaziland. Journal of Arid Environments, 74(6), 675–684.

[ece39268-bib-0055] Trollope, W. S. W. , Potgieter, A. L. F. , & Zambatis, N. (1989). Assessing veld condition in the Kruger National Park using key grass species. Koedoe, 32(1), 67–93.

[ece39268-bib-0056] van de Koppel, J. , Rietkerk, M. , & Weissing, F. J. (1997). Catastrophic vegetation shifts and soil degradation in terrestrial grazing systems. Trends in Ecology & Evolution, 12(9), 352–356. 21238102 10.1016/s0169-5347(97)01133-6

[ece39268-bib-0057] Verweij, R. J. T. , Verrelst, J. , Loth, P. E. , Heitkönig, I. M. A. , & Brunsting, A. M. H. (2006). Grazing lawns contribute to the subsistence of mesoherbivores on dystrophic savannas. Oikos, 114(1), 108–116.

[ece39268-bib-0058] Waldram, M. S. , Bond, W. J. , & Stock, W. D. (2008). Ecological engineering by a mega‐grazer: White rhino impacts on a south African savanna. Ecosystems, 11(1), 101–112.

[ece39268-bib-0059] Wang, Y. , Naumann, U. , Eddelbuettel, D. , & Warton, D. (2018). mvabund: Statistical methods for analysing multivariate abundance data . R package version 3.13.1. https://CRAN.R‐project.org/package=mvabund

[ece39268-bib-0060] Wigley, B. J. , Charles‐Dominique, T. , Hempson, G. P. , Stevens, N. , TeBeest, M. , Archibald, S. , Bond, W. J. , Bunney, K. , Coetsee, C. , Donaldson, J. , Fidelis, A. , Gao, X. , Gignoux, J. , Lehmann, C. , Massad, T. J. , Midgley, J. J. , Millan, M. , Schwilk, D. , Siebert, F. , … Kruger, L. M. (2020). A handbook for the standardised sampling of plant functional traits in disturbance‐prone ecosystems, with a focus on open ecosystems. Australian Journal of Botany, 68(8), 473–531.

[ece39268-bib-0061] Yoganand, K. , & Owen‐Smith, N. (2014). Restricted habitat use by an African savanna herbivore through the seasonal cycle: Key resources concept expanded. Ecography, 37(10), 969–982.

