# Supplementary material for: Grazing lawns and overgrazing in frequently grazed grass communities
Source: Ecol Evol. 2022 Sep 13;12(9):e9268. doi: 10.1002/ece3.9268 (PMC9468907; doi:10.1002/ece3.9268)
Supplement: Supplementary file 1 — Tables S1–S7 [file ECE3-12-e9268-s001.docx]

**Appendix 1**

**Grazing lawns and overgrazing in frequently grazed grass communities**

Gareth P. Hempson^1,2*^, Catherine L. Parr^1,3,4^, Caroline E. R. Lehmann^1,5,6^ & Sally Archibald^1^

*^1^Centre for African Ecology, School of Animal, Plant and Environmental Sciences, University of the Witwatersrand, Johannesburg, 2050, South Africa.*

*^2^South African Environmental Observation Network (SAEON), Ndlovu Node, Phalaborwa, Kruger National Park, 1390, South Africa.*

*^3^Department of Earth, Ocean & Ecological Sciences, University of Liverpool, Liverpool, L69 3GP, UK.*

*^4^Department of Zoology & Entomology, University of Pretoria, Private Bag X20, Pretoria 0028, South Africa.*

*^5^School of GeoSciences, University of Edinburgh, Edinburgh, EH9 3FF, United Kingdom.*

*^6^Tropical Diversity, Royal Botanic Garden Edinburgh, Edinburgh, EH3 5NZ, United Kingdom.*

* Corresponding author: Gareth P. Hempson (ghempson@gmail.com)

Contact address: School of Animal, Plant and Environmental Sciences, University of the Witwatersrand, 1 Jan Smuts Ave, Braamfontein, Johannesburg, South Africa

Table S1: Study site descriptions

Table S2: Grass species, growth form and trait data

Table S3: Grass species trait PCA eigenvalues and variable loadings

Table S4: Grass growth form classification v.test values

Table S5: Environmental variable PCA eigenvalues and variable loadings

Table S6: AICc values for multiple regression analyses of percentage bare ground in lateral attractor-dominated grass communities

Table S7: AICc values for multiple regression analyses of percentage bare ground in tufted attractor-dominated grass communities

Table S1: Study site descriptions including sampling date, geographic co-ordinates, mean annual rainfall (MAR), altitude, site type (communal = communal grazing area), sampling area dimensions (size) and number of plots (total with rows and columns in brackets), and the region and country (RSA = South Africa, TNZ = Tanzania) in which sites were located.

| Site | Date | Latitude | Longitude | MAR (mm) | Altitude (m) | Type | Size (m) | No. plots | Region & country |
| --- | --- | --- | --- | --- | --- | --- | --- | --- | --- |
|  |  |  |  |  |  |  |  |  |  |
| Berg-en-Dal | 2015-03-25 | -25.43272 | 31.44813 | 576 | 345 | Soccer field | 52 x 100 | 30 (5 x 6) | Kruger NP, Mpumalanga, RSA |
| Black Rock | 2014-12-11 | -32.73741 | 28.29242 | 466 | 22 | Natural | 60 x 100 | 30 (6 x 5) | Eastern Cape, RSA |
| Camdeboo | 2015-02-20 | -32.24210 | 24.46139 | 336 | 892 | Natural | 44 x 66 | 30 (5 x 6) | Eastern Cape, RSA |
| Cape Vidal | 2015-01-13 | -28.14290 | 32.54379 | 844 | 11 | Soccer field | 40 x 90 | 30 (5 x 6) | KwaZulu-Natal, RSA |
| Commando Drift | 2014-12-09 | -32.07892 | 26.01527 | 381 | 1033 | Natural | 60 x 100 | 30 (6 x 5) | Eastern Cape, RSA |
| Gariep Dam | 2015-02-18 | -30.55680 | 25.61916 | 339 | 1271 | Natural | 60 x 100 | 30 (5 x 6) | Free State, RSA |
| Giants Castle | 2015-01-29 | -29.19329 | 29.57950 | 743 | 1450 | Communal | 60 x 100 | 30 (6 x 5) | KwaZulu-Natal, RSA |
| Golden Gate | 2015-02-14 | -28.46862 | 28.74754 | 716 | 1774 | Natural | 60 x 100 | 30 (5 x 6) | Free State, RSA |
| Great Fish | 2014-12-06 | -32.97438 | 26.87284 | 431 | 409 | Natural | 60 x 100 | 30 (6 x 5) | Eastern Cape, RSA |
| iMfolozi | 2015-01-20 | -28.28487 | 31.95863 | 833 | 183 | Airstrip | 32 x 100 | 30 (5 x 6) | KwaZulu-Natal, RSA |
| Ithala | 2015-01-22 | -27.50420 | 31.20176 | 596 | 579 | Soccer field | 60 x 80 | 30 (6 x 5) | KwaZulu-Natal, RSA |
| Kirawira | 2016-07-26 | -2.18157 | 34.22785 | 987 | 1214 | Natural | 24 x 48 | 20 (4 x 5) | Serengeti NP, TNZ |
| Kogatende | 2016-07-20 | -1.56696 | 34.91681 | 949 | 1425 | Natural | 24 x 48 | 20 (4 x 5) | Serengeti NP, TNZ |
| Koppies Dam | 2015-02-12 | -27.22017 | 27.71311 | 508 | 1408 | Natural | 60 x 60 | 15 (5 x 3) | Free State, RSA |
| Lower Sabie | 2015-03-24 | -25.12293 | 31.92035 | 483 | 179 | Soccer field | 60 x 80 | 30 (5 x 6) | Kruger NP, Mpumalanga, RSA |
| Luchaba | 2014-12-12 | -31.54667 | 28.72995 | 681 | 725 | Communal | 60 x 100 | 30 (6 x 5) | Eastern Cape, RSA |
| Mara | 2016-07-21 | -1.55893 | 34.85780 | 949 | 1405 | Natural | 24 x 48 | 20 (4 x 5) | Serengeti NP, TNZ |
| Marakele | 2015-03-04 | -24.53246 | 27.48324 | 449 | 1012 | Natural | 60 x 100 | 30 (5 x 6) | Limpopo Province, RSA |
| Maria Moroka | 2015-02-17 | -29.30272 | 26.89900 | 507 | 1522 | Natural | 60 x 100 | 30 (5 x 6) | Free State, RSA |
| Mkambati | 2014-12-14 | -31.29071 | 30.01199 | 789 | 19 | Natural | 60 x 100 | 30 (6 x 5) | Eastern Cape, RSA |
| Mokala | 2015-02-22 | -29.10580 | 24.38734 | 394 | 1174 | Natural | 60 x 100 | 30 (5 x 6) | Northern Cape, RSA |
| Mountain Zebra | 2015-02-19 | -32.21735 | 25.42884 | 351 | 1412 | Natural | 60 x 100 | 30 (5 x 6) | Eastern Cape, RSA |
| Mpofu | 2014-12-07 | -32.61766 | 26.59473 | 398 | 956 | Natural | 60 x 100 | 30 (6 x 5) | Eastern Cape, RSA |
| Ndumo | 2015-01-16 | -26.91446 | 32.29866 | 456 | 143 | Airstrip | 36 x 100 | 30 (5 x 6) | KwaZulu-Natal, RSA |
| Pilanesburg | 2015-03-03 | -25.24716 | 27.13423 | 457 | 1159 | Natural | 60 x 100 | 30 (5 x 6) | North West, RSA |
| Satara | 2015-03-23 | -24.39978 | 31.78067 | 368 | 259 | Soccer field | 60 x 120 | 30 (5 x 6) | Kruger NP, Mpumalanga, RSA |
| Seronera | 2016-07-25 | -2.43241 | 34.85969 | 627 | 1546 | Natural | 24 x 48 | 20 (4 x 5) | Serengeti NP, TNZ |
| Soit | 2016-07-29 | -2.58190 | 35.14319 | 448 | 1707 | Natural | 24 x 48 | 20 (4 x 5) | Serengeti NP, TNZ |
| Spioenkop | 2015-02-13 | -28.71606 | 29.51189 | 731 | 1151 | Soccer field | 60 x 100 | 30 (5 x 6) | KwaZulu-Natal, RSA |
| St Lucia | 2015-01-12 | -28.35865 | 32.41606 | 962 | 17 | Soccer field | 60 x 80 | 30 (6 x 5) | KwaZulu-Natal, RSA |
| Tembe | 2015-01-15 | -27.02109 | 32.49357 | 490 | 49 | Airstrip | 36 x 125 | 30 (5 x 6) | KwaZulu-Natal, RSA |
| Tsolwana | 2014-12-08 | -32.18542 | 26.51173 | 433 | 1653 | Natural | 48 x 125 | 30 (5 x 6) | Eastern Cape, RSA |
| Willem Pretorius | 2015-02-15 | -28.28893 | 27.24728 | 438 | 1398 | Natural | 60 x 100 | 30 (5 x 6) | Free State, RSA |
|  |  |  |  |  |  |  |  |  |  |

Table S2: Grass species information including the number of plot-level records across all sites, which countries the species occurred in, growth form and whether this was determined by clustering or manual assessment (G. P. Hempson), and the four trait values. Note that the three species with no culm orientation index did not have any culm material in specimens encountered within the study plots.

| Species | Plot-level records | Country | Growth form | Method | Lateral index | Tuft index | Culm orientation index | Grazer use  index |
| --- | --- | --- | --- | --- | --- | --- | --- | --- |
|  |  |  |  |  |  |  |  |  |
| *Aristida adscensionis* | 1 | RSA | Avoider | Manual | 0.00 | 0.00 | 0.00 | 0.11 |
| *Aristida bipartita* | 5 | RSA | Tufted attractor | Manual | 0.00 | 1.00 | 0.88 | 0.93 |
| *Aristida congesta* | 75 | RSA | Tufted attractor | Clustering | 0.01 | 0.53 | 0.64 | 0.83 |
| *Aristida junciformis junciformis* | 17 | RSA | Avoider | Clustering | 1.00 | 0.00 | 1.00 | 0.70 |
| *Aristida scabrivalis contracta* | 10 | RSA | Tufted attractor | Manual | 0.00 | 0.10 | 0.59 | 0.91 |
| *Aristida* sp. unknown | 1 | TNZ | Avoider | Manual | 0.00 | 0.00 | 1.00 | 0.50 |
| *Bothriochloa insculpta* | 29 | RSA, TNZ | Tufted attractor | Clustering | 0.48 | 0.86 | 0.32 | 0.88 |
| *Brachiaria eruciformis* | 3 | RSA | Tufted attractor | Manual | 0.00 | 0.00 | 0.67 | 0.83 |
| *Cenchrus ciliaris* | 3 | RSA | Avoider | Manual | 0.33 | 0.67 | 0.25 | 0.48 |
| *Chloris gayana* | 14 | RSA, TNZ | Lateral attractor | Clustering | 0.93 | 0.07 | 0.27 | 0.95 |
| *Chloris pycnothrix* | 1 | TNZ | Avoider | Manual | 0.00 | 0.00 | 0.75 | 0.67 |
| *Chloris virgata* | 23 | RSA | Tufted attractor | Clustering | 0.17 | 0.04 | 0.34 | 0.95 |
| *Chrysochloa orientalis* | 28 | TNZ | Avoider | Clustering | 0.96 | 0.00 | 0.85 | 0.81 |
| *Cymbopogon marginatus* | 1 | RSA | Avoider | Manual | 1.00 | 0.00 | 1.00 | 0.24 |
| *Cymbopogon pospischilii* | 7 | RSA | Resister | Manual | 0.00 | 1.00 | 1.00 | 0.00 |
| *Cynodon dactylon* | 220 | RSA | Lateral attractor | Clustering | 1.00 | 0.00 | 0.38 | 0.90 |
| *Cynodon incompletus* | 169 | RSA | Lateral attractor | Clustering | 0.97 | 0.01 | 0.26 | 0.83 |
| *Cynodon nlemfluensis* | 8 | TNZ | Lateral attractor | Manual | 1.00 | 0.00 | 0.09 | 0.82 |
| *Dactyloctenium australe* | 62 | RSA | Lateral attractor | Clustering | 0.95 | 0.00 | 0.10 | 0.84 |
| *Digitaria didactyla* | 26 | RSA | Avoider | Clustering | 1.00 | 0.00 | 0.13 | 0.53 |
| *Digitaria eriantha* | 8 | RSA | Lateral attractor | Manual | 0.88 | 0.13 | 0.14 | 0.91 |
| *Digitaria longiflora* | 2 | RSA | Lateral attractor | Manual | 1.00 | 0.00 | 0.25 | 0.83 |
| *Digitaria macroblephara* | 20 | TNZ | Tufted attractor | Clustering | 0.65 | 0.35 | 0.60 | 0.78 |
| *Digitaria natalensis* | 10 | RSA | Tufted attractor | Manual | 0.20 | 0.80 | 0.50 | 1.00 |
| *Digitaria ternata* | 2 | RSA | Avoider | Manual | 0.50 | 0.00 | 0.00 | 0.52 |
| *Elionuris muticus* | 1 | RSA | Resister | Manual | 0.00 | 1.00 | 0.75 | 0.02 |
| *Eragrostis capensis* | 4 | RSA | Tufted attractor | Manual | 0.00 | 0.50 | 0.50 | 0.92 |
| *Eragrostis chloromelas* | 62 | RSA | Tufted attractor | Clustering | 0.52 | 0.47 | 0.75 | 0.97 |
| *Eragrostis cilianensis* | 8 | RSA | Avoider | Manual | 0.00 | 0.00 | 0.38 | 0.69 |
| *Eragrostis ciliaris* | 3 | RSA | Resister | Manual | 0.00 | 0.67 | 0.50 | 0.29 |
| *Eragrostis curvula* | 88 | RSA | Tufted attractor | Clustering | 0.30 | 0.70 | 0.48 | 0.94 |
| *Eragrostis gummiflua* | 13 | RSA | Resister | Clustering | 0.00 | 1.00 | 0.80 | 0.89 |
| *Eragrostis heteromera* | 17 | RSA | Resister | Clustering | 0.00 | 1.00 | 0.66 | 0.74 |
| *Eragrostis lehmanniana* | 46 | RSA | Tufted attractor | Clustering | 0.13 | 0.87 | 0.45 | 1.00 |
| *Eragrostis obtusa* | 17 | RSA | Tufted attractor | Clustering | 0.47 | 0.41 | 0.47 | 0.84 |
| *Eragrostis patentipilosa* | 6 | RSA | Avoider | Manual | 0.00 | 1.00 | 0.46 | 0.09 |
| *Eragrostis plana* | 63 | RSA | Resister | Clustering | 0.00 | 1.00 | 0.93 | 0.48 |
| *Eragrostis planiculmis* | 1 | RSA | Resister | Manual | 0.00 | 1.00 | 1.00 | 0.66 |
| *Eragrostis racemose* | 25 | RSA | Resister | Clustering | 0.04 | 0.92 | 0.56 | 0.69 |
| *Eragrostis rigidior* | 6 | RSA | Tufted attractor | Manual | 0.33 | 0.83 | 0.17 | 0.83 |
| *Eragrostis* sp. unknown | 1 | RSA | Resister | Manual | 0.00 | 1.00 | - | 0.42 |
| *Eragrostis superba* | 84 | RSA | Tufted attractor | Clustering | 0.00 | 0.96 | 0.42 | 0.80 |
| *Eragrostis tenuifolia* | 23 | TNZ | Resister | Clustering | 0.00 | 1.00 | 0.90 | 0.69 |
| *Eragrostis trichophora* | 37 | RSA | Lateral attractor | Clustering | 0.70 | 0.03 | 0.27 | 0.94 |
| *Eustachys paspaloides* | 15 | TNZ | Lateral attractor | Clustering | 1.00 | 0.00 | 0.55 | 0.94 |
| *Harpachne schimperi* | 20 | TNZ | Tufted attractor | Clustering | 0.00 | 1.00 | 0.43 | 0.83 |
| *Harpochloa falx* | 2 | RSA | Resister | Manual | 0.00 | 0.00 | - | 0.48 |
| *Helictotrichon turgidulum* | 1 | RSA | Avoider | Manual | 0.00 | 1.00 | 0.00 | 0.36 |
| *Heteropogon contortus* | 55 | RSA | Tufted attractor | Clustering | 0.00 | 0.93 | 0.18 | 0.89 |
| *Hyparrhenia hirta* | 14 | RSA | Tufted attractor | Clustering | 0.64 | 0.36 | 0.58 | 0.78 |
| *Imperata cylindrical* | 13 | RSA | Avoider | Clustering | 1.00 | 0.00 | 1.00 | 0.42 |
| *Melica decumbens* | 10 | RSA | Resister | Manual | 0.00 | 0.00 | 1.00 | 0.20 |
| *Microchloa caffra* | 28 | RSA | Resister | Clustering | 0.25 | 0.61 | 0.68 | 0.57 |
| *Microchloa kunthii* | 9 | RSA, TNZ | Resister | Manual | 0.11 | 0.89 | 0.58 | 0.38 |
| *Panicum aequinerve* | 18 | RSA | Avoider | Clustering | 0.67 | 0.00 | 0.21 | 0.63 |
| *Panicum coloratum* | 15 | RSA | Lateral attractor | Clustering | 1.00 | 0.00 | 0.15 | 0.97 |
| *Panicum maximum* | 1 | RSA | Tufted attractor | Manual | 0.00 | 0.00 | 0.00 | 0.68 |
| *Paspalum dilatatum* | 4 | RSA | Tufted attractor | Manual | 0.50 | 0.50 | 0.08 | 0.59 |
| *Paspalum notatum* | 2 | RSA | Lateral attractor | Manual | 1.00 | 0.00 | 0.13 | 0.47 |
| *Paspalum scrobiculatum* | 40 | RSA | Lateral attractor | Clustering | 0.80 | 0.00 | 0.28 | 0.90 |
| *Pennisetum clandestinum* | 1 | RSA | Lateral attractor | Manual | 1.00 | 0.00 | 0.00 | 0.00 |
| *Pennisetum mezianum* | 8 | TNZ | Avoider | Manual | 1.00 | 0.00 | 0.78 | 0.36 |
| *Perotis patens* | 30 | RSA | Tufted attractor | Clustering | 0.33 | 0.23 | 0.41 | 0.93 |
| *Sacciolepis curvata* | 2 | RSA | Tufted attractor | Manual | 0.50 | 0.50 | 0.50 | 0.97 |
| *Schmidtia pappophoroides* | 11 | RSA | Tufted attractor | Clustering | 0.55 | 0.18 | 0.40 | 0.84 |
| *Setaria sphacelata* | 17 | RSA | Lateral attractor | Clustering | 0.94 | 0.06 | 0.09 | 0.81 |
| *Setaria verticillata* | 3 | RSA | Avoider | Manual | 0.00 | 0.00 | 0.83 | 0.71 |
| *Sporobolus africanus* | 79 | RSA | Resister | Clustering | 0.10 | 0.82 | 0.86 | 0.56 |
| *Sporobolus fimbriatus* | 30 | RSA, TNZ | Resister | Clustering | 0.60 | 0.43 | 0.92 | 0.59 |
| *Sporobolus ioclados* | 87 | RSA, TNZ | Lateral attractor | Clustering | 0.93 | 0.02 | 0.44 | 0.76 |
| *Sporobolus nitens* | 29 | RSA | Tufted attractor | Clustering | 0.07 | 0.93 | 0.46 | 0.99 |
| *Sporobolus pyramidalis* | 27 | RSA | Resister | Clustering | 0.00 | 1.00 | 1.00 | 0.41 |
| *Sporobolus virginicus* | 21 | RSA, TNZ | Lateral attractor | Clustering | 1.00 | 0.00 | 0.50 | 0.76 |
| *Stenotaphrum secundatum* | 78 | RSA | Lateral attractor | Clustering | 1.00 | 0.00 | 0.02 | 0.69 |
| *Themeda triandra* | 144 | RSA, TNZ | Tufted attractor | Clustering | 0.19 | 0.80 | 0.45 | 0.90 |
| *Trachypogon spicatus* | 1 | RSA | Avoider | Manual | 1.00 | 0.00 | 1.00 | 0.53 |
| *Tragus berteronianus* | 45 | RSA | Avoider | Clustering | 0.11 | 0.00 | 0.45 | 0.53 |
| *Tragus koelerioides* | 47 | RSA | Avoider | Clustering | 0.74 | 0.00 | 0.21 | 0.40 |
| *Trichoneura grandiglumis* | 6 | RSA | Avoider | Manual | 0.00 | 1.00 | 0.04 | 0.42 |
| *Tristachya leucothrix* | 1 | RSA | Avoider | Manual | 0.00 | 1.00 | 0.00 | 0.43 |
| Unknown species 1 | 1 | RSA | Avoider | Manual | 0.00 | 0.00 | - | 0.64 |
| Unknown species 2 | 15 | RSA | Lateral attractor | Manual | 1.00 | 0.00 | 0.50 | 0.98 |
| Unknown species 3 | 15 | RSA | Tufted attractor | Manual | 0.00 | 1.00 | 1.00 | 0.85 |
| Unknown species 4 | 6 | RSA | Avoider | Manual | 1.00 | 0.00 | 0.08 | 0.42 |
| Unknown species 5 | 5 | RSA | Avoider | Manual | 1.00 | 0.00 | 0.60 | 0.57 |
| *Urochloa mossambicensis* | 53 | RSA | Tufted attractor | Clustering | 0.38 | 0.47 | 0.28 | 0.92 |
| *Urochloa oligotricha* | 10 | RSA | Tufted attractor | Manual | 0.10 | 0.00 | 0.30 | 0.86 |
| *Urochloa panicoides* | 28 | RSA | Avoider | Clustering | 0.04 | 0.00 | 0.71 | 0.59 |
|  |  |  |  |  |  |  |  |  |

Table S3: Eigenvalues, percentage variance and trait loadings from the principal component analysis of grass species traits performed prior to hierarchical clustering to identify grass growth forms.

|  | PC1 | PC2 | PC3 | PC4 |
| --- | --- | --- | --- | --- |
|  |  |  |  |  |
| Eigenvalue | 2.086 | 1.222 | 0.518 | 0.174 |
| % of variance | 52.2 | 30.5 | 13.0 | 4.3 |
| Cumulative % of variance | 52.2 | 82.7 | 95.7 | 100.0 |
|  |  |  |  |  |
| *Trait loadings:* |  |  |  |  |
| Lateral index | -0.886 | -0.296 | 0.219 | 0.282 |
| Tuft index | 0.886 | 0.349 | -0.063 | 0.299 |
| Culm orientation index | 0.668 | -0.510 | 0.540 | -0.042 |
| Grazer use index | -0.266 | 0.868 | 0.418 | -0.049 |
|  |  |  |  |  |

Table S4: Grass trait contributions to defining growth form clusters, where v.tests are used to compare trait values within clusters to the overall trait values.

| Growth form & trait | v.test | Category mean | Overall mean | Category std. dev. | Overall std. dev. | P value |
| --- | --- | --- | --- | --- | --- | --- |
|  |  |  |  |  |  |  |
| Lateral attractors |  |  |  |  |  |  |
| *Lateral index* | 4.491 | 0.935 | 0.492 | 0.089 | 0.393 | 0.000 |
| *Grazer use index* | 1.988 | 0.857 | 0.774 | 0.086 | 0.167 | 0.047 |
| *Culm orientation index* | -3.300 | 0.275 | 0.496 | 0.162 | 0.267 | 0.001 |
| *Tuft index* | -3.723 | 0.016 | 0.393 | 0.024 | 0.404 | 0.000 |
|  |  |  |  |  |  |  |
| Avoiders |  |  |  |  |  |  |
| *Tuft index* | -2.994 | 0.000 | 0.393 | 0.000 | 0.404 | 0.003 |
| *Grazer use index* | -3.625 | 0.577 | 0.774 | 0.128 | 0.167 | 0.000 |
|  |  |  |  |  |  |  |
| Tufted attractors |  |  |  |  |  |  |
| *Grazer use index* | 3.487 | 0.887 | 0.774 | 0.068 | 0.167 | 0.000 |
| *Tuft index* | 2.562 | 0.595 | 0.393 | 0.299 | 0.404 | 0.010 |
| *Lateral index* | -2.664 | 0.288 | 0.492 | 0.227 | 0.393 | 0.008 |
|  |  |  |  |  |  |  |
| Resisters |  |  |  |  |  |  |
| *Culm orientation index* | 3.914 | 0.812 | 0.496 | 0.139 | 0.267 | 0.000 |
| *Tuft index* | 3.863 | 0.865 | 0.393 | 0.197 | 0.404 | 0.000 |
| *Grazer use index* | -2.980 | 0.623 | 0.774 | 0.137 | 0.167 | 0.003 |
| *Lateral index* | -3.211 | 0.110 | 0.492 | 0.190 | 0.393 | 0.001 |
|  |  |  |  |  |  |  |

Table S5: Eigenvalues, percentage variance and variable loadings from the principal component analysis of environmental variables for all study sites.

|  | PC1 | PC2 | PC3 | PC4 |
| --- | --- | --- | --- | --- |
|  |  |  |  |  |
| Eigenvalue | 2.062 | 1.000 | 0.773 | 0.164 |
| % of variance | 51.6 | 25.0 | 19.3 | 4.1 |
| Cumulative % of variance | 51.6 | 76.6 | 95.9 | 100.0 |
|  |  |  |  |  |
| *Variable loadings:* |  |  |  |  |
| Mean annual rainfall | -0.472 | -0.632 | 0.614 | -0.028 |
| Percent sand | -0.612 | 0.666 | 0.391 | 0.170 |
| Cation exchange capacity | 0.922 | -0.181 | 0.184 | 0.289 |
| pH | 0.785 | 0.352 | 0.458 | -0.224 |
|  |  |  |  |  |

Table S6: ΔAICc values for multiple regression models of percent bare ground in relation to percent sand, mean annual rainfall and dung for sites dominated by lateral-attractor grasses.

| Model | d.f. | AICc | ΔAICc |
| --- | --- | --- | --- |
|  |  |  |  |
| Intercept only | 2 | 159.959 | 0 |
| Sand | 3 | 160.794 | 0.834 |
| Rain | 3 | 161.547 | 1.588 |
| Dung | 3 | 162.870 | 2.910 |
| Rain + Sand | 4 | 162.915 | 2.956 |
| Dung + Sand | 4 | 163.978 | 4.019 |
| Dung + Rain | 4 | 164.882 | 4.923 |
| Dung + Rain + Sand | 5 | 166.409 | 6.450 |
| Rain × Sand | 5 | 166.803 | 6.844 |
| Dung × Sand | 5 | 167.880 | 7.921 |
| Dung × Rain | 5 | 168.788 | 8.828 |
| Dung × Sand + Rain | 6 | 171.003 | 11.044 |
| Dung × Rain + Sand | 6 | 171.039 | 11.080 |
| Rain × Sand + Dung | 6 | 171.042 | 11.082 |
| Dung × Sand + Rain × Sand | 6 | 176.566 | 16.606 |
| Dung × Rain + Dung × Sand | 7 | 176.566 | 16.607 |
| Dung × Rain + Rain × Sand | 7 | 176.603 | 16.644 |
| Dung × Rain + Dung × Sand + Rain × Sand | 8 | 183.365 | 23.406 |
| Dung × Rain × Sand | 9 | 191.864 | 31.905 |
|  |  |  |  |

Table S7: ΔAICc values for multiple regression models of percent bare ground in relation to percent sand, mean annual rainfall and dung for sites dominated by tufted-attractor grasses.

| Model | d.f. | AICc | ΔAICc |
| --- | --- | --- | --- |
|  |  |  |  |
| Dung | 3 | 128.867 | 0.000 |
| Intercept only | 2 | 129.982 | 1.115 |
| Rain | 3 | 131.407 | 2.539 |
| Dung + Sand | 4 | 131.874 | 3.007 |
| Sand | 3 | 132.245 | 3.378 |
| Dung + Rain | 4 | 132.302 | 3.434 |
| Rain + Sand | 4 | 134.650 | 5.782 |
| Dung × Sand | 5 | 135.440 | 6.573 |
| Dung + Rain + Sand | 5 | 136.306 | 7.439 |
| Dung × Rain | 5 | 136.462 | 7.595 |
| Rain × Sand | 5 | 136.748 | 7.880 |
| Rain × Sand + Dung | 6 | 137.629 | 8.761 |
| Dung × Sand + Rain | 6 | 141.003 | 12.135 |
| Dung × Rain + Sand | 6 | 141.852 | 12.985 |
| Dung × Rain + Rain × Sand | 7 | 144.037 | 15.169 |
| Dung × Sand + Rain × Sand | 7 | 144.918 | 16.051 |
| Dung × Rain + Dung × Sand | 7 | 147.860 | 18.993 |
| Dung × Rain + Dung × Sand + Rain × Sand | 8 | 153.917 | 25.049 |
| Dung × Rain × Sand | 9 | 167.816 | 38.949 |
|  |  |  |  |
